# The Hurricane Lung: A Case of Hydrocarbon Pneumonitis With Abscess Formation Following Fuel Siphoning

**DOI:** 10.7759/cureus.14807

**Published:** 2021-05-02

**Authors:** Renuka Reddy, Jessica Baek, Hanna R Perone, Kai Chen, Daniel M Lichtstein

**Affiliations:** 1 Internal Medicine, University of Miami JFK Medical Center, Atlantis, USA; 2 Internal Medicine, University of Miami Miller School of Medicine, Miami, USA

**Keywords:** fuel siphoning, hydrocarbon pneumonitis, abscess, hurricane, gasoline

## Abstract

Fuel siphoning is a widespread practice worldwide, but infrequently observed in the United States. Some reports suggest greater incidence of fuel siphoning during the hurricane season. Fuel siphoning is associated with a high risk of hydrocarbon toxicity, often leading to the development of hydrocarbon pneumonitis. This form of exogenous lipoid pneumonia can present acutely with chest pain and dyspnea. While most cases of hydrocarbon pneumonitis resolve spontaneously with supportive care, rarely patients develop life-threatening complications. We present the case of a 56-year-old man who developed hydrocarbon pneumonitis complicated by abscess formation after attempting to siphon fuel from a gasoline tank in preparation for a hurricane.

## Introduction

In the United States, accidental ingestion of petroleum gasoline has largely been limited to the pediatric population and adult street performers, namely, fire eaters. Fuel siphoning is another form of accidental gasoline ingestion that is a relatively common practice in developing countries, but is less frequently seen in the United States [1]. However, a higher incidence of fuel siphoning has been reported during hurricane season due to scarce resources [2]. Fuel siphoning involves forceful suction through a tube, which can lead to large volume aspiration [1].

Accidental ingestion or aspiration of gasoline can result in hydrocarbon pneumonitis, which is a form of exogenous lipoid pneumonia [3]. Those who siphon fuel are at a high risk for developing pneumonitis due to the low surface tension, low viscosity, and high volatility of hydrocarbons, which facilitate mucosal spread [4,5]. In the lungs, hydrocarbons disrupt surfactant, decrease pulmonary compliance, and cause bronchial edema with resultant tissue damage to the lung parenchyma [1,6,7].

Aspirated oil particles impair mucociliary clearance and fail to stimulate the cough reflex, thereby allowing them to reach the lower respiratory tract [8]. Lipid hydrocarbons concentrate within alveoli and are unable to be degraded due to a lack of specific enzymes in humans [3]. Hydrocarbons provoke an inflammatory response leading to activation of macrophages which phagocytose the lipids [6]. The lipids may remain within macrophages for prolonged periods, but are released back into the alveoli after macrophages lyse [3]. This lipid release may trigger a subsequent giant cell response resulting in fibrosis [3].

Clinical manifestations of hydrocarbon pneumonitis are often non-specific and depend on various factors, such as frequency of exposure, type and quantity of aspirated material, and body posture during the inciting incident [8]. Therefore, patients may have a wide spectrum of symptoms ranging from gradual but progressive worsening of dyspnea, fever, and cough to acute severe respiratory distress and chest pain [3]. Most reported cases, whether acute or insidious, had favorable clinical outcomes without significant morbidity or mortality [1]. Here, we describe a case of severe hydrocarbon pneumonitis complicated by lung abscess.

## Case presentation

A 56-year-old man presented to the emergency department with pleuritic chest pain and four episodes of hemoptysis hours after attempting to siphon fuel from a gasoline tank with his mouth [9]. His past medical history was remarkable for hypertension and type II diabetes mellitus. On initial presentation, respiratory rate was 30 breaths per minute, heart rate was 130 beats per minute, blood pressure was 196/102 mmHg, and oxygen saturation was 91% on room air. Physical examination was notable for normal oral mucosa with increased lacrimation, patent airway, diminished breath sounds with bilateral rales, and no accessory muscle use [9]. He was alert and oriented without any focal neurologic deficits. He was immediately placed on a non-rebreather mask.

**Figure 1:**
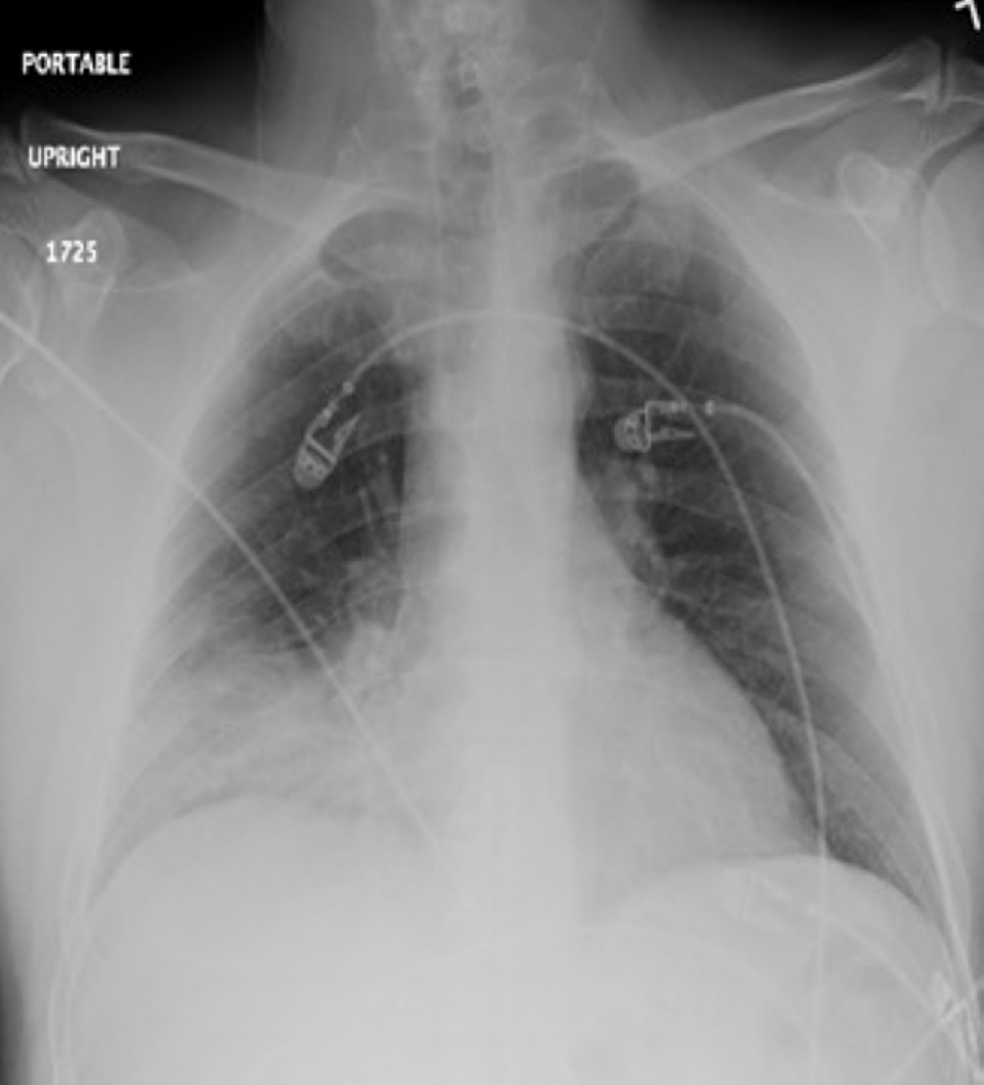
Chest radiograph demonstrating right middle and lower lobe infiltrates.

**Figure 2:**
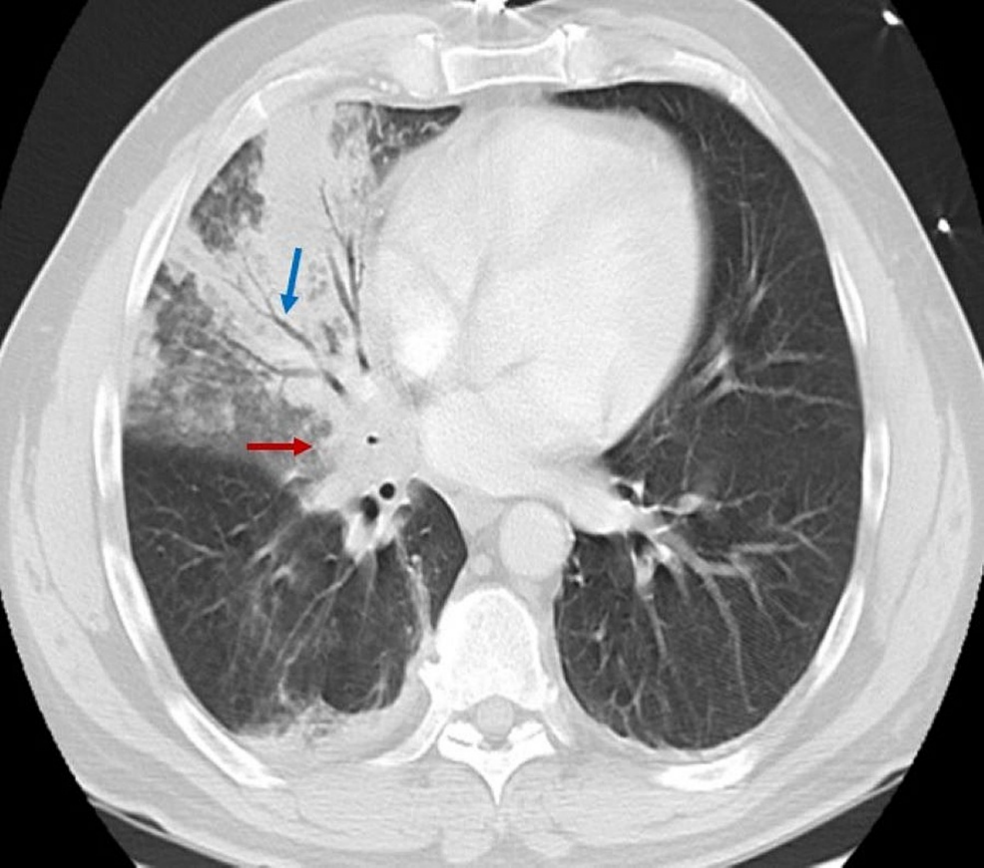
Chest CT depicting patchy airspace disease with air bronchograms (blue arrow) in the right middle lobe and a right infrahilar mass (red arrow) measuring 3.3 × 2.6 cm with encasement of the right middle lobe bronchi. CT: computed tomography

The white blood cell count was 11.2 109/L. Arterial blood gas revealed the following: pH 7.410, pCO_2_ 37.2 mmHg, pO_2_ 104 mmHg on 100% FiO_2_. Chest radiograph demonstrated right middle and lower lobe infiltrates (Figure 1) [9]. Chest computed tomography (CT) with contrast showed patchy airspace disease and air bronchograms in the aforementioned areas with a right infrahilar mass measuring 3.3 × 2.6 cm near the right middle lobe bronchi (Figure 2) [9].

He was admitted to the intensive care unit, ultimately requiring intubation and mechanical ventilation. Sputum and blood cultures drawn on admission revealed no growth. A repeat chest radiograph demonstrated the development of a pleural effusion, felt to be parapneumonic. A thoracentesis was performed with removal of 300 cc of yellow-colored fluid [9]. Pleural fluid analysis was exudative with a negative gram stain and culture. Bronchoscopy demonstrated friable mucosa in the bronchi of the right middle and lower lobes [9]. Purulent fluid was noted, which was suggestive of abscess formation. Gram stain of the aspirated fluid was remarkable for white blood cells without organisms.

The patient underwent thoracotomy with right middle lobe resection [9]. Surgical pathology revealed extensive acute and chronic inflammation with lipoid droplets, extensive necrosis, and obliteration of alveolar spaces. These findings were consistent with the diagnosis of necrotizing lipoid pneumonia with abscess formation following hydrocarbon aspiration. The patient’s hospitalization was further complicated by an acute cerebellar stroke. He required tracheostomy and percutaneous endoscopic gastrostomy tube placement. He was ultimately discharged to a long-term acute care hospital.

## Discussion

Patients typically present with hydrocarbon pneumonitis acutely within a few hours of fuel siphoning [1]. Symptoms often include cough, chest pain, dyspnea, hypoxia, and low-grade fever [1,6]. CT of the chest commonly shows necrotic air-space consolidations in the right middle lobe, with varying involvement of the bilateral lower lobes [1,6,7]. The predilection for the right middle lobe is thought to be due to the squatting position assumed by patients while siphoning [3,7]. In addition to consolidation, chest CT may also reveal ground-glass opacifications, air-space nodules, and crazy-paving pattern (ground glass attenuation with superimposed septal thickening) [1,7]. Hydrocarbon pneumonitis is definitively diagnosed by bronchoscopy, with findings of lipid-laden macrophages on bronchoalveolar lavage [1,6].

Most accounts of hydrocarbon pneumonitis are generally mild to moderate in severity. Management is largely supportive with most cases resolving spontaneously. Treatment regimens have included antibiotics, glucocorticoids, and bronchoalveolar lavage, although there is insufficient evidence to suggest any therapeutic benefits [1]. Symptoms often subside within five to seven days [8]. Radiographic opacities resolve between two weeks to eight months, although these changes are normally preceded by clinical improvement [5,6]. Most patients recover without long-term sequela [1,6].

While the majority of incidents of hydrocarbon pneumonitis are self-limited, severe cases, such as the one depicted here, may require extensive management. Patients presenting with significant hypoxia may necessitate non-invasive positive pressure ventilation or intubation and mechanical ventilation [1,10]. On rare occasions, hydrocarbon pneumonitis may be complicated by the development of empyema, pneumothorax, bronchopulmonary fistula formation, or acute respiratory distress syndrome [1,7,10].

Another rare complication of hydrocarbon pneumonitis is abscess formation, as observed in this case. To the best of our knowledge, there are only a small number of reported cases of hydrocarbon aspiration complicated by pulmonary abscess [1]. The risk factors for lung abscess formation in hydrocarbon pneumonitis remain unknown. However, the lung parenchyma is disrupted by both the direct toxic effects of hydrocarbon on the capillaries, alveolar septa, and pulmonary epithelium, as well as the indirect effects on surfactant solubility and production [11]. Evidence from a study in rats exposed to hydrocarbon particles also suggests that destruction of the antioxidant defense system induces cellular dysfunction and tissue damage, resulting in pulmonary edema and hemorrhagic necrosis [12]. Although rare, these severe complications can be fatal without timely diagnosis and treatment.

## Conclusions

While fuel siphoning is relatively common worldwide, cases of fuel siphoning in the United States have largely been associated with natural disasters, namely, hurricanes. It is crucial for clinicians to be mindful of this etiology, as hydrocarbon pneumonitis may mimic bacterial pneumonia and delay potential interventions. Fuel siphoning is distinct from chronic repeated occupational exposures because siphoning exposes the patient to higher volumes of gasoline due to the suction utilized. This practice may result in rapid symptom onset and more severe disease. Early diagnosis is essential to mitigate the development of potentially fatal complications.
